# The effect of movement representation techniques on ankle function and performance in persons with or without a lateral ankle sprain: a systematic review and meta-analysis

**DOI:** 10.1186/s12891-023-06906-9

**Published:** 2023-10-04

**Authors:** Luuk J.F. Siemes, Maarten P. van der Worp, P. Henk J.A. Nieuwenhuijzen, Niki M. Stolwijk, Thomas Pelgrim, J. Bart Staal

**Affiliations:** 1 School of Sport and Exercise, HAN University of Applied Sciences, Nijmegen, the Netherlands; 2 School of Allied Health, HAN University of Applied Sciences, Nijmegen, the Netherlands; 3 HAN Study Centres, HAN University of Applied Sciences, Nijmegen, the Netherlands; 4 School of Allied Health, HAN University of Applied Sciences and IQ Healthcare, Radboud Institute for Health Sciences, Radboud University Medical Center, Nijmegen, the Netherlands

**Keywords:** Imagery, Psychotherapy, Systematic review [Publication Type], Lateral ligament, Ankle, Rehabilitation, Muscle strength

## Abstract

**Background:**

Lateral ankle sprains are highly prevalent and result in tissue damage, impairments of muscle strength, instability, and muscle activation. Up to 74% will experience ongoing symptoms after a lateral ankle sprain. In healthy subjects, motor imagery might induce neural changes in the somatosensory and motor areas of the brain, yielding favourable enhancements in muscular force. However, during motor imagery, difficulties in building a motor image, no somatosensory feedback, and the absence of structural changes at the level of the muscle might explain the differences found between motor imagery and physical practice. In rehabilitation, motor imagery might be supportive in rebuilding motor networks or creating new networks to restore impairments in muscle activation and movement patterns. This systematic review was undertaken to summarize the current body of evidence about the effect on motor imagery, or action observation, on lower leg strength, muscle performance, ankle range of motion, balance, and edema in persons with, and without, a lateral ankle sprain compared to usual care, a placebo intervention, or no intervention.

**Methods:**

A systematic review with meta-analysis of randomized controlled trials was conducted in healthy participants and participants with a lateral ankle sprain. Motor imagery or action observation in isolation, or in combination with usual care were compared to a placebo intervention, or no intervention. An electronic search of MEDLINE, EMBASE, Cinahl, Psychinfo, Sportdiscus, Web of Science, Cochrane and Google Scholar was conducted, and articles published up to 7^th^ June 2023 were included. Two reviewers individually screened titles and abstracts for relevancy using the inclusion criteria. Variables related to muscle strength, muscle function, range of motion, balance, return to sports tests, or questionnaires on self-reported function or activities were extracted. A risk of bias assessment was done using the Cochrane Risk-of-Bias tool II by two reviewers. Meta-analysis using a random effects model was performed when two or more studies reported the same outcome measures. The Standardized Mean Difference (SMD) was calculated over the change from baseline scores. Review manager 5.4 was used to perform analysis of subgroup differences and test for statistically significant differences. Confidence intervals were visually checked for overlap between subgroups.

**Results:**

Nine studies, six examining healthy participants and three examining participants with an acute lateral ankle sprain, were included. All studies were rated with moderate to high risk of bias overall. Quality of the motor imagery interventions differed largely between studies. Meta-analysis showed a large and significant effect of motor imagery on lower leg strength (SMD 1.47, 95% CI 0.44 to 2.50); however, the evidence was downgraded to very low certainty due to substantial heterogeneity (I^2^ = 73%), limitations in the studies (some concerns in risk of bias in all studies), and imprecision (*n* =  < 300). Evidence showed no association with ankle range of motion (SMD 0.25, 95% CI -0.43 to 0.93), edema (SMD -1.11, 95% CI -1.60 to 3.81), the anterior reach direction of the Star Excursion Balance Test (SEBT) (SMD 0.73, 95% CI -0.62 to 2.08), the posterolateral direction (SMD 0.32, 95% CI -0.94 to 1.57), and the posteromedial direction (SMD 0.52, 95% CI -0.07 to 1.10). The certainty of evidence for the different comparisons was very low.

**Conclusions:**

There is a low certainty, significant, positive effect for motor imagery being able to improve lower leg muscle strength in healthy participants. The effect on balance, range of motion and edema was uncertain and of very low certainty.

**Systematic review registration:**

PROSPERO CRD42021243258.

**Supplementary Information:**

The online version contains supplementary material available at 10.1186/s12891-023-06906-9.

## Introduction

Lateral ankle sprains are highly prevalent in the general population and during sports activities [1, 2]. It is estimated that 20% to 40% of all sports injuries are ankle sprains [3] resulting in considerable time loss from sports activities [4]. On average, 15 to 28 days being lost was reported in amateur and professional soccer players [5, 6]. In particular, athletes involved in basketball, volleyball and field sports are at increased risk of sustaining an ankle sprain [7]. Lateral ankle sprains are often classified as a one-time injury, yet evidence suggests that up to 74% of persons with a lateral ankle sprain will experience persistent symptoms [8]. There is evidence for a multifactorial contribution of impaired balance, reaction time and strength to the development of ankle instability [9]. Further, lateral ankle sprains might result in instability and impairments in strength, peroneal muscle activation, proprioception, range of motion, joint laxity and decreased self-reported functioning [10–12]. The initial sprain results in tissue damage, but additionally causes impairments in the sensory-perceptual and motor-behavioural systems [12]. For instance, participants with ankle instability were found to activate the muscles of the hip, knee and ankle later than control participants without ankle instability during a shift from double leg stance to single leg stance [13]. The impaired movement patterns have been related to changes within the central nervous system [14] due to functional reorganization of the cortex, increasing the contribution of secondary sensorimotor areas [15]. Brain areas responsible for generating movement are activated extensively during the execution of movement, but similarly also during observation and imagination of the same movement [16–18].

Movement representation techniques can be defined as “the representation of movement, especially observation and/or imagination of normal pain-free movement” [19]. Motor imagery and action observation are two extensively studied movement representation techniques [20] and are widely used in a sport and in a rehabilitation context [21]. Motor imagery is defined as a cognitive and dynamic ability involving the cerebral representation of an action, without its real motor execution. Action observation training is considered as the internal representation of a set of movements evoked by the observer during live visualization of the movements [22]. Positive effects on motor performance, such as movement speed, accuracy, variability, and muscle strength, have been demonstrated in athletes [23]. In addition, training-mode specific effects were observed in a study examining the effect of six weeks of motor imagery training in professional basketball players. A group mentally rehearsing back squat and bench press exercises at 85% one repetition maximum (1-RM) had greater maximal strength gains, whereas the optimum power loads group experienced greater improvements on lower limb jumping capacity and muscular power [24]. Moreover, motor imagery showed beneficial effects on maximal muscle voluntary strength in healthy adults, but when compared to physical practice, a small benefit in favour of physical practice was found [25]. Motor imagery might induce neural changes in the somatosensory and motor areas of the brain, yielding favourable enhancements in muscular force. However, during motor imagery, difficulties in building a motor image, no somatosensory feedback, and the absence of structural changes at the level of the muscle might explain the differences found between motor imagery and physical practice [24, 25].

In rehabilitation, motor imagery might be supportive in rebuilding motor networks or creating new networks to restore impairments in muscle activation and movement patterns [17] and is of specific interest in a setting where movement is impaired due to pain or injury and could provide a substitute to actual exercise [22, 26]. Positive effects on muscle activation, muscle strength and functional outcomes have been demonstrated in persons after a total knee arthroplasty [27] and anterior cruciate ligament reconstruction [28].

Over the last twenty years, several studies have looked at the effect of motor imagery on ankle function and performance in healthy participants [10, 29] and after an ankle injury [30–32]. However, there is a considerable heterogeneity in the methodology and interventions studied, for instance the type and duration of motor imagery. To date, no systematic review has been undertaken into the topic and there is a need for a clear overview of the effect of motor imagery on ankle function and performance. Therefore, the goal of this systematic review was to summarize the current body of evidence. Our research question was:

What is the effect of motor imagery or action observation on ankle function (muscle strength, range of motion, edema) and performance (balance, return to sport tests) in persons with, or without, a lateral ankle sprain?

## Methods

This systematic review was performed in accordance with the guideline Preferred Reporting Items for Systematic reviews and Meta-Analysis: The PRISMA statement [33] and was prospectively registered (PROSPERO CRD42021243258).

### Identification and selection of studies

An electronic search was conducted using the databases MEDLINE, EMBASE, Cinahl, Psychinfo, Sportdiscus, Web of Science, Cochrane and Google Scholar. The search strategy was designed in consultation with two experienced librarians [TP and DM]. Articles published up to 7^th^ June 2023 were included in the search. The results were uploaded to Rayyan [34], and duplicate results were removed.

Medical Subject Headings (MeSH) and free-text words were used to design the search string. Boolean operator “AND” was used to combine keywords and “OR” was used to combine the keywords within each group. The following search terms were used and combined to search the databases: motor, mental, movement, mirror, imagery, ankle, injuries. The final searches for all databases can be found in Appendix 1. The electronic search was conducted by one librarian [TP] and two reviewers [LS and MW] screened titles and abstracts for relevancy using the inclusion criteria [33, 35] randomized controlled trials published in peer-reviewed journals. Selection of studies was limited to articles written in English, German or Dutch. Trials including healthy participants, or participants with a lateral ankle sprain were included. No restrictions to age were made. The intervention consisted of movement representation techniques (motor imagery or action observation) in isolation, or in combination with usual care. Whereas the control group received usual care, an intervention which differed from the movement representation techniques, a placebo intervention, or did not receive any intervention. Studies were included if measured outcome measures related to function or performance, such as muscle strength, muscle endurance, range of motion, balance, return to sports tests, or questionnaires on self-reported function or activities were reported.

If disagreement upon article relevancy existed between the two reviewers, consensus was achieved by discussion. Full texts of eligible articles were retrieved and evaluated individually by the two reviewers. Again, disagreements were resolved using discussion to reach consensus. A third reviewer [HN] was consulted to make the final decision if disagreement remained during the process. Additionally, reference lists of included articles were checked manually to identify additional literature. The Kappa statistic and percentage of agreement as a measure for interrater reliability were calculated and Kappa results were interpreted as: 0.01–0.20 none to slight, 0.21–0.40 fair 0.41–0.60 moderate, 0.61–0.80 substantial, and 0.81–1.00 almost perfect agreement.

### Assessment of characteristics of studies

#### Study quality

A risk of bias assessment was conducted blinded and individually by two reviewers [LS and MW]. The Cochrane risk-of-bias tool 2 for randomized trials was used to judge the domains: bias arising from the randomization process; bias due to deviations from intended interventions; bias due to missing outcome data; bias in measurement of the outcome and bias in selection of the reported result [36]. Upon disagreement, consensus was reached through discussion. A third reviewer [HN] was available in case of persisting disagreement. The risk of bias judgement was presented in a table alongside the results of the systematic review [37].

### Data analysis

The data collection process was performed using a data extraction form [38]. The items were discussed by the reviewer and colleagues [LS, MW, HN, NS, and JBS] in order to develop the form. The extraction form was pilot-tested by two reviewers on three randomly-articles and the extraction form were revised where needed. The primary reviewer [LS] extracted the information and the second reviewer [MW] checked the extracted results. Disagreement was settled using discussion. If agreement could not be reached, a third reviewer [HN] was consulted to provide the final decision [33]. The final version of the data extraction form can be found in Appendix 3. The following key information was extracted: author(s), year of publication, origin/country of origin, the aim of the study, inclusion and exclusion criteria, demographic description of the population (gender, age, type of injury, duration of injury) and sample size, the methodology, the intervention (detailed information about the design, the application and theory), duration of the intervention, the comparison group, the outcomes measures (i.e. muscle strength, muscle endurance, range of motion, edema, functional tests, questionnaires), the measurement instruments and their validity and reliability, the follow-up (and duration), and key findings.

Meta-analysis using a random effects model was performed when two, or more, studies reported the same outcome measures in a comparable study population. Statistical heterogeneity of the intervention effect was assessed using the I^2^ as it is preferred over the Chi-square test and was considered not important if I^2^ was between 0 and 30%; moderate between 30 and 50%, substantial between 50 and 75%, and considerable above 75% [39]. Review Manager (4.5) [40] and SPSS, version 28, were used to prepare and maintain the systematic review and conduct the meta-analysis. Outcomes on a continuous scale were presented as the weighted average using the mean difference (MD) and standard deviation. The standardized mean difference (SMD) and standard deviations were presented if studies used different instruments to measure the same construct. The MD or SMD were calculated over the change from baseline scores [41], authors were contacted if the published results were insufficient for the data analysis. When possible, subgroup analysis to explore whether the results differed between healthy subject and injured participants was performed. A statistical test (Borenstein and Higgins, 2013) to explore for subgroup differences was conducted using Review Manager 5.4 when a minimum of 10 studies were included in the analysis. Since there were only two subgroups, a visual exploration of differences was performed by checking if confidence intervals were overlapping.

The trial registers ClinicalTrials.gov, EU Clinical Trials Register, Netherlands Trial Register, WHO International Clinical Trials Registry Platform (ICTRP) and ISRCTN registry were checked to identify unpublished trials.

The qualitative analysis of the body of evidence was performed using the Grading of Recommendations, Assessment, Development and Evaluation (GRADE) framework. The certainty of evidence was classified as high, moderate, low, or very low certainty. Evidence from randomized controlled trials began as high certainty evidence yet could be downgraded based on concerns in any of the following five categories: risk of bias, imprecision, inconsistency, indirectness, and publication bias [42–45].

#### Quality of the motor imagery intervention

The quality of the motor imagery interventions was analysed using fifteen predefined criteria by Schuster et al. [46]. Motor imagery interventions might differ in type, duration, and context. Differences in the administration of the intervention might produce different results, and these differences might be responsible for variation in the estimates of effect between the studies [47].

Motor imagery sessions were scored successful when: (1) performed individually; (2) added after physical practice; (3) were supervised; (4) not directed; (5) the location of the motor imagery and (6) position of the participants was task-specific; (7) accompanied by acoustic and (8) detailed MI instructions; (9) performed with the eyes closed; (10) the perspective used during MI was an internal view with (11) kinaesthetic mode and (12) MI interventions included primarily motor-focused activities. (13) The average duration of a study was around 34 days, (14) with a total of 3 MI training sessions per week, and (15) had an average duration of 17 min per training session (with a minimum of 178 min) [46].

## Results

### Flow of studies through the review

**Fig. 1:**
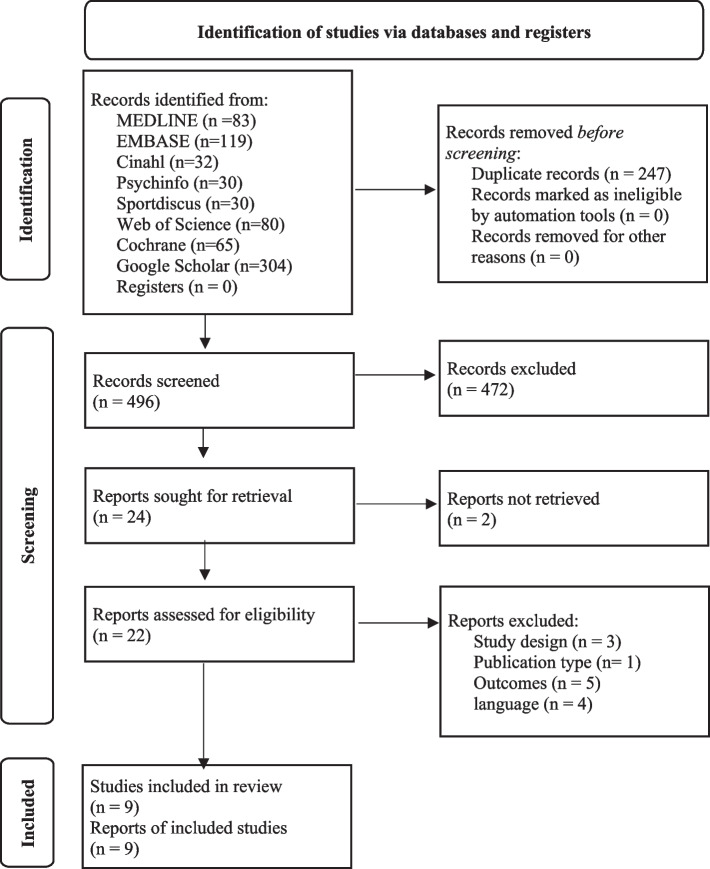
Flow diagram of the identification of studies

An electronic search of the databases identified 743 records (see Fig. 1; MEDLINE 83 records, EMBASE 119 records, Cinahl 32 records, Psychinfo 30 records, Sportdiscus 30 records, Web of Science 80 records, Cochrane 65 records, and Google Scholar 304 records). After removing duplicates, 496 records were screened for title and abstract. In total, 472 records were excluded based on study design, population, outcome, or intervention. The Kappa statistic for agreement between reviewers after screening titles and abstracts was 0.738 (substantial agreement) and the percentage of agreement was 97,7%. From the 24 included reports, 22 full text articles were retrieved, two reports led to ongoing studies and could therefore not be retrieved. Thirteen papers were excluded based on study design (no control group, *n* = 3) [10, 48, 49], outcomes (no outcomes on ankle function or performance, *n* = 5), [50–54] language (Korean, *n* = 2; Arabic, *n* = 2) [55–58], or publication type (not peer reviewed, *n* = 1) [59]. The Kappa statistic for agreement after screening full texts was 0.68 (substantial agreement), and the percentage of agreement was 98%. The third reviewer was consulted once to aid in decision making, further disputes were solved through careful deliberation and consensus was reached. In total, nine randomized controlled trials were included [29–32, 60–64]. Reference lists were checked manually for relevant studies, no additional reports were identified.

### Characteristics of studies

#### Risk of bias

Risk of bias assessment revealed at least some concerns in six studies [29, 30, 60, 61, 63, 64] and high concerns in three [31, 32, 62] of the included studies (see Table 1). All studies used a random allocation sequence. Due to the nature of the intervention, in all studies participants and researchers delivering the intervention were probably aware of the assignment during the trial. In two studies a blinded assessor performed the pre-intervention measurements of range of motion, postural control, swelling and functional instability; however, the post-intervention measurements were not conducted by a blinded assessor [30, 63]. The other seven studies lacked information about the blinding of the assessor during the measurements [29, 31, 32, 60–62, 64]. Risk of bias assessments were conducted per outcome measure, as recommended by the Cochrane Handbook for Systematic Reviews [65]. Results of the risk of bias assessments were presented per study when the result for outcomes were equal. For the study of Nagar & Noohu [60] a small difference in risk of bias between the outcome measures was observed and these results were presented individually (see Table 1).

**Table 1:** Risk of bias assessment

Study ID	Experimental	Comparator	Outcome	D1	D2	D3	D4	D5	Overall
**Abraham et al. **[63]	Motor imagery	Upper body exercises	Range of Motion tests	+	+	+	-	!	!
**Bouguetoch et al. **[64]	Motor imagery	Control group	Maximal voluntary contraction	+	+	+	+	!	!
**Christakou et al.** [32]	Physiotherapy + motor imagery	Physiotherapy	Muscular endurance, functional stability, dynamic balance	+	+	+	-	!	-
**Christakou & Zervas **[31]	Physiotherapy + motor imagery	Physiotherapy	Range of Motion, swelling	+	+	+	-	!	-
**Dehghan et al.** [62]	Motor imagery + proprioceptive exercise	Control groups	Proprioception	+	-	-	-	!	-
**Grosprêtre et al. **[61]	Motor imagery	Control group	Muscle torque	+	+	+	!	!	!
**Nagar & Noohu **[60]	Strength + balance + motor imagery	Strength + balance exercises	1RM ankle plantar flexion strength	+	+	+	+	!	!
**Nagar & Noohu **[60]	Strength + balance + motor imagery	Strength + balance exercises	Balance (mSEBT)	+	+	+	!	!	!
**Nunes et al. **[30]	Physiotherapy + motor imagery	Physiotherapy	Ankle ROM, balance (mSEBT), swelling, functional instability questionnaire	+	+	+	!	!	!
**Sidaway & Trzaska** [29]	Motor imagery	Exercise	Maximal dorsal flexion torque	+	+	+	+	!	!

+ low risk, ! some risk,—high risk, *D1* randomization process, *D2* deviations from the intended interventions, *D3* missing outcome data, *D4* measurement of the outcome, *D5* selection of the reported result, *1RM* 1 repetition maximum, *ROM* range of motion, *mSEBT* modified star balance excursion test

One trial protocol was identified, no further (published) pre-analysis plans were discovered, increasing risk of bias in the ‘selection of the reported result’ domain [30]. Five trial registers (ClinicalTrials.gov, EU Clinical Trials Register, Netherlands Trial Register, WHO International Clinical Trials Registry Platform (ICTRP) and ISRCTN registry) were checked to study non-reporting bias and in total, 334 results were located using the search criteria ‘ankle’ and ‘motor imagery’. Two trials in participants with lateral ankle sprain were identified, one recent trial is in the recruiting phase, and one has been included in the current systematic review. Publication bias was not assessed by testing for funnel plot asymmetry due to insufficient number of studies (*n* =  < 10) included per meta-analysis [66].

#### Participants and sample sizes

The characteristics of the nine included studies can be found in Table 2. Three studies [30–32] recruited athletes with an acute ankle sprain and six studies recruited healthy participants [29, 60–64]. Participants in the studies were aged 13 to 15 years [63], 16 to 18 years [62], 16 to 20 years [30] and 18 to 30 years [29, 31, 32, 60, 61, 64]. The sample sizes in the studies varied from 18 [31, 61] to 400 participants [64]. The study of Deghan et al. [62] recruited 16 football squads (*n* = 400; 25 players per squad). The article does not provide detailed information about the intervention in both groups, the outcome measures and which measurement instruments were used. Data extraction was limited to participant information. Most studies divided participants into an intervention group and a control group, except for two studies [29, 64], one used a three-arm-design [29] and one used three intervention groups and one control group [64]. When assigned to the groups, sample sizes were between 7 [64] and 25 [62] participants per group.

**Table 2:** Characteristics of included studies

Trial	Participants (age and complaints)	Motor imagery intervention description; setting	Control	Outcome measures	Follow-up	Results
**Abraham et al. (2017)** [63]	Sample of 25 active female dance students with a minimum of three years dance experience Intervention group (*n* = 13) aged 13.51 ± 0.49 and control group (*n* = 12) aged 13.63 ± 0.52	Two elevé tasks, a repeat (10 repetitions of 3 s) and static (hold 10 s at maximum height) task were imagined for 20 to 25 min per session. 2 sessions per week for 6 weeks delivered as a group session in the dance studio	Upper body exercises (postural awareness, mobility, and strength) while seated, 2 times per week for 6 weeks	1) Maximal ankle plantarflexion angle 2) plantarflexion ROM 3) symmetry index	Post-intervention	No significant differences between groups during pre-measurements. MI improved repeat task in maximal plantarflexion angle (*p* = .04) and ROM (*p* = .02) for the intervention group during post-measurements. No statistically significant results on ankle plantarflexion maximal angle or ROM were noted in the static task
**Bouguetoch et al. (2021)** [64]	Sample of 37 (aged 24 ± 5.8 years) healthy participants, 12 females, were recruited. Motor imagery group (*n* = 10, 4 females), control group (*n* = 7, 2 females), NMES + MI group (*n* = 10, 3 females), and NMES group (*n* = 10, 3 females)	10 MI training sessions (5 per week) under supervision in the laboratory. 40 maximal isometric plantar flexion movements of the right leg were imagined over 6 s, with 6 s rest. Participants were seated on the isokinetic ergometer during the training sessions	The control group did not perform any exercise Additional intervention groups: The NMES group underwent 40 evoked contractions of the triceps surae muscles and the NMES + MI group underwent 20 imagined and 20 evoked contractions	1) isometric torque 2) EMG activity 3) Muscle architecture	Post-intervention	A significant improvement was demonstrated for the NMES and MI group, as compared to the control group (*p* < 0.001 and *p* = 0.0023) and NMES + MI group (*p* = 0.004 and *p* = 0.002) There was no significant difference between the NMES and MI group (*p* = 0.934)
**Christakou et al. (2007)** [32]	20 participants with a grade II ankle sprain in sports (± 5.0 ± 2.4 days after trauma) aged 25.4 ± 4.76 years (range 18–30)	Participants first underwent 60 min of normal physiotherapy and continued with visualization of ankle dorsal- and plantarflexion ROM, strengthening exercises, proprioceptive training, stationary cycling, forward lunges, step-ups and -downs, diagonal hops and stretching exercises for 45 min. In total 12 sessions, 3 times per week. Motor imagery was done while seated in a quiet place	12 sessions of 60 min physiotherapy with hydromassage, laser and exercises: ankle dorsal- and plantarflexion ROM, strengthening exercises, proprioceptive training, stationary cycling, forward lunges, step-ups and -downs, diagonal hops and stretching exercises. 3 times per week	1) muscular endurance 2) functional stability 3) dynamic balance	Post-intervention	The MI group showed significantly greater muscular endurance (*p* = .017) compared to the control group. No other significant differences between the experimental and control group were observed using Bonferroni corrections
**Christakou & Zervas (2007)** [31]	18 athletes with grade II sprain (5 ± 2.49 days after trauma) aged 26 ± 4.47 years (range 18 to 30 years) and at least 2 years of athletic experience	Participants underwent relaxation and imagery for 45 min in addition to 60 min physiotherapy. A relaxation technique was used before MI, participants sat on a quiet place and imagined the exercises as conducted during the physiotherapy session as instructed by the investigator	12 sessions of 60 min physiotherapy including hydro-massage, ultrasound, laser, and exercises: ankle ROM, strengthening, proprioceptive, stationary cycling, step-ups and down, diagonal hops and stretching	1) VAS 2) swelling 3) ROM	Post-intervention	The results did not show a significant difference between the experimental and control group and between the measurements and the intervention group versus control group on pain (*p* = .74), edema (*p* = .78) and ROM (*p* = .78)
**Dehghan et al. (2013)** [62]	16 sports teams with 25 healthy football players per squad (*n* = 400) aged 16 to 18 years were recruited	Mental and proprioceptive exercise for 6 months	No mental and proprioceptive exercises	1) proprioception 2) Number of ankle inversion traumas	Post-intervention	Proprioceptive and MI exercises improved proprioception (*p* = .000) and reduced the number of injuries (*p* = .027) compared to the control group
**Grosprêtre et al. (2017)** [61]	18 healthy young adults not involved in intense sport activities were recruited. Participants in the intervention group (n = 9) were aged 22.2 ± 2.6 years and in the control group (n = 9) 23.2 ± 2.8 years	4 series of 25 imagined maximal isometric contractions of the plantar flexors of the right leg were carried out seated and strapped on an isokinetic dynamometer, daily during seven days for 20 min per session	No exercise during the study (one week)	1) isometric torque 2) rate of torque development	Post-intervention	A significant interaction effect between time and group was found on MVC torques (*p* = .024). Only the MI group significantly improved MVC torque. A significant interaction was found between time and group for EMG activity in the M. soleus (*p* = .039) and M. gastrocnemius caput medialis (*p* = .024)
**Nagar & Noohu (2014)** [60]	30 healthy, male, collegiate basketball players were recruited. The intervention group (*n* = 25) was aged 20.53 ± 2.2 years and participants in the control group (*n* = 15) were aged 20.80 ± 2.4 years	Mental imagery followed directly after the strength and balance training and took 10 min. Participant sat in a quiet place and performed breathing exercises for 2 min, followed by 3 sets of imagination of the previous performed strength and balance exercises with a 1-min rest The intervention was carried out 3 times per week for 6 weeks	30 min of balance board exercises: double-leg stance, side to side balance with eyes open and closed and functional sports activities on 1 leg. Strength training with leg press, 4 sets building up to 6RM. 3 times per week for 6 weeks	1) balance 2) strength ankle flexors	Post-intervention	There was no significant effect of the intervention as compared to the control group for the m-SEBT in the anterior direction (*p* = .118), posteromedial direction (*p* = .169), the posterolateral direction (*p* = .303), and for the composite score (*p* = .22) There was a significant effect for knee extensor strength (*p* = .023) in favour of the intervention group and no significant effect for ankle plantar flexor strength (*p* = .052)
**Nunes et al. (2015)** [30]	18 male, field soccer players, with a recent ankle sprain (< 72 h). The intervention group was aged 17.2 ± 1.6 years and the control group was aged 17.4 ± 1.8 years (range: 16—20 years)	Motor imagery after physical therapy treatment. In a quiet room participants sat in front of a computer. 40 different images of ankle–foot were projected on screen, patient had to identify a left or right ankle using the left–right arrows of the keyboard within 4 s. Time and number of right guesses was calculated at the end of the session. Intervention took place5 times per week, 2 h per session (physical therapy) plus motor imagery (< 2:40 min.)	Physical therapy treatment: cryotherapy (20 min) in first 2 sessions, electrotherapy (TENS, ultrasound, or laser) in first sessions, kinesiotherapy consisting of stretching, joint mobilization (passive and active), sensorimotor training, strengthening exercises, and return to sport exercises in the final phase. Sessions were 5 times per week and took 2 h	1) ROM 2) Postural control 3) Swelling 4) Functional instability	Post-intervention	No significant between-group differences were demonstrated for ankle dorsal flexion ROM (*p* = .23), plantar flexion ROM (*p* = .50), the m-SEBT anterior direction (*p* = .70), the posterolateral direction (*p* = .29), the posteromedial direction (*p* = .79), edema (*p* = .50), and the CAIT (*p* = .70)
**Sidaway & Trzaska (2005)** [29]	24 healthy participants were recruited and allocated to 3 groups of 8 participants: MI, EG and CG The mean age was 22.7 years (range 19 to 26 years)	Participants in the mental and physical practice group performed 3 sets of 10 repetitions of maximal dorsiflexion contraction while seated on Biodex for 3 times per week, 4 weeks, 15 min per session	Participants did not participate in any form of practice during the 4 weeks of the experiment	1) maximal dorsiflexion torque	Post-intervention	ANOVA revealed a significant main effect for group (*p* = < .01). Post-test performance on dorsiflexion peak torque was significantly higher in mental and physical practice group, not in control group (*p* = < .05). There was no significant difference between the improvement for the mental practice and the physical practice group

*CAIT* Cumberland Ankle Instability Tool, *CG* control group, *EG* exercise group, *IG* intervention group, *MI* motor imagery, *m-SEBT* modified Star Excursion Balance Test, *MVC* maximal voluntary contraction, *NMES* neuromuscular electrical stimulation, *RCT* randomized controlled trial, *ROM* range of motion, *VAS* visual analogue scale

#### Description of the intervention and control groups

In the studies using injured athletes, the control groups received normal physiotherapy and the intervention groups received normal physiotherapy and an additional motor imagery intervention [30–32]. The study with healthy dancers compared a motor imagery intervention with upper body exercises [63], and the study with healthy basketball players compared strength training, balance training and motor imagery to a control group receiving strength training and balance training [60]. In one study, motor imagery was compared to a control group which did not perform any exercise [61] and in the study of Sidaway & Trzaska [29], participants were randomly divided into a motor imagery group, an exercise group and a control group. The study using four groups compared differences between a motor imagery group, neuromuscular electrical stimulation group, a group combining motor imagery with neuromuscular electrical stimulation, and a control group [64]. One study performed “a proprioception training with mental imagery” and compared it to a control group. However, it is unclear what the intervention exactly encompassed and how it was delivered as there is no description provided in the article about the content of the mental imagery intervention, duration, or frequency of the intervention [62].

#### Quality and content of the motor imagery

An assessment of the quality of the motor imagery intervention based on the criteria of Schuster et al. [46] can be found in Table 3. Most of the studies directed the motor imagery using a verbal, read-aloud, prespecified protocol [29, 31, 32, 60, 63], one study combined it with an audio file [61], and in one study ankle–foot images were presented on a computer [30]. In some studies, the intervention took less than 17 min per session [60] or did not reach a total of 178 min during the intervention period [61] or failed to satisfy both criteria (volume per session and in total) [30]. Both studies from Christakou & Zervas and Christakou et al. included 12 motor imagery sessions lasting 45 min [31, 32]. The study of Nunes et al. [30] scored no, no information, or not applicable on 12 out of 15 criteria. This was largely due to the nature of the intervention: participants in the motor imagery intervention were shown 40 left–right images (lateralization) in contrast to the movement-related visualization given in most other studies. For the study of Dehghan et al. [62], no information was provided on any of the items in the article.

**Table 3:** Characteristics and analysis of the quality of the motor imagery interventions

	**Criteria of successful motor imagery interventions**	**Abraham et al. (2017) **[63]	**Bouguetoch et al. (2021) **[64]	**Christakou et al. (2007) **[32]	**Christakou & Zervas (2007) **[31]	**Deghan et al. (2013) **[62]	**Grospretre et al. (2017) **[61]	**Nagar & Noohu (2014) **[60]	**Nunes et al. (2015) **[30]	**Sidaway & Trzaska (2005) **[29]
**1**	Were performed individual;	N	Y	Y	Y	NI	Y	Y	Y	Y
**2**	Were added after physical practice;	N	N	Y	Y	NI	N	Y	Y	N
**3**	Were supervised;	Y	Y	NI	Y	NI	Y	Y	NI	Y
**4**	Were not directed;	N	N	N	N	NI	N	N	N	N
**5 & 6**	The location of the MITS and position of the participants were task-specific;	Y	Y	Y	Y	NI	Y	Y	N	Y
**7 & 8**	The participants received acoustic and detailed MI instructions;	Y	Y	NI	Y	NI	Y	Y	N	Y
**9**	During MI the eyes were closed;	Y	NI	NI	NI	NI	NI	NI	N	Y
**10—12**	The used perspective during MI was an internal view with kinaesthetic mode and MI interventions included primarily motor-focused activities;	Y	Y	Y	Y	NI	Y	Y	N	Y
**13**	The average duration was 34 days;	Y	N	Y	Y	NI	N	Y	NI	N
**14**	With a total of 3 MI training sessions per week;	N	Y	Y	Y	NI	Y	Y	Y	Y
**15**	And had an average duration of 17 min per training session (total minimum 178 min)	NI	N	Y	Y	NI	N	N	N	Y
	Total score (items rated ‘Yes’)	10	10	10	13	0	10	12	3	12

*N* no, *Y* yes, *NI* no information, *N/A* not applicable, criteria based on Schuster et al. [46]

#### Motor imagery ability

Three studies used the Movement Imagery Questionnaire-Revised (MIQ-R) and one the Movement Imagery Questionnaire-Revised second version (MIQ-RS) to assess the individual’s ability to image four movements using internal visual imagery, and kinaesthetic imagery [60, 61, 63, 64]. In addition, one used a predetermined item score of ≥ 4 to include participants for their study [60]. The MIQ-R uses 8 items (4 visual and 4 kinaesthetic), and the MIQ-RS uses 14 items (7 visual and 7 kinaesthetic). Participants first form a visual image of a movement, and secondly feel what performing this movement is like. Their effort is rated on a 1 (very hard to see/feel) to 7 (very easy to see/feel) scale. The MIQ-R has a maximum of 56 points, where a higher score correlates with a better motor imagery ability, and the MIQ-RS a maximum of 49 points for both visual and kinaesthetic motor ability.

Grosprêtre et al. [61] reported 46.9 ± 3.5 points (maximum score: 56), Bouguetoch et al. [64] 46.1 ± 6.1 points and Nagar & Noohu [60] calculated the average for each item (5 ± 0.84 and 5.07 ± 0.91; maximum score: 7). Abraham et al. [63] calculated their scores per imagery type and participants scored 22.92 ± 3.14 points for visual and 20.31 (± 4.55) for kinaesthetic motor imagery.

In the two studies of Christakou et al. [31, 32] the Vividness of Movement Imagery Questionnaire (VMIQ) was used. The VMIQ uses 24-items on a 5-point scale from 1 (perfectly clear and vivid as normal vision to 5 (no image at all, you only “know” that you are thinking of the skill) to rate motor imagery ability for ‘doing it yourself’ and to image ‘somebody else’ do it. A score of 24 is the highest possibility and 120 the lowest. Christakou et al. [31, 32] report 66 ± 14.53 points for “watching somebody else” and 57.89 ± 14.24 points for “doing himself/herself” in one study and 65.80 ± 13.72 points for “watching somebody else” and 56.00 ± 14.70 points for “doing himself/herself” in their other study. Three studies did not assess, or report on the imagery ability of the participants [29, 30, 62].

### Effect of intervention

**Fig. 2:**
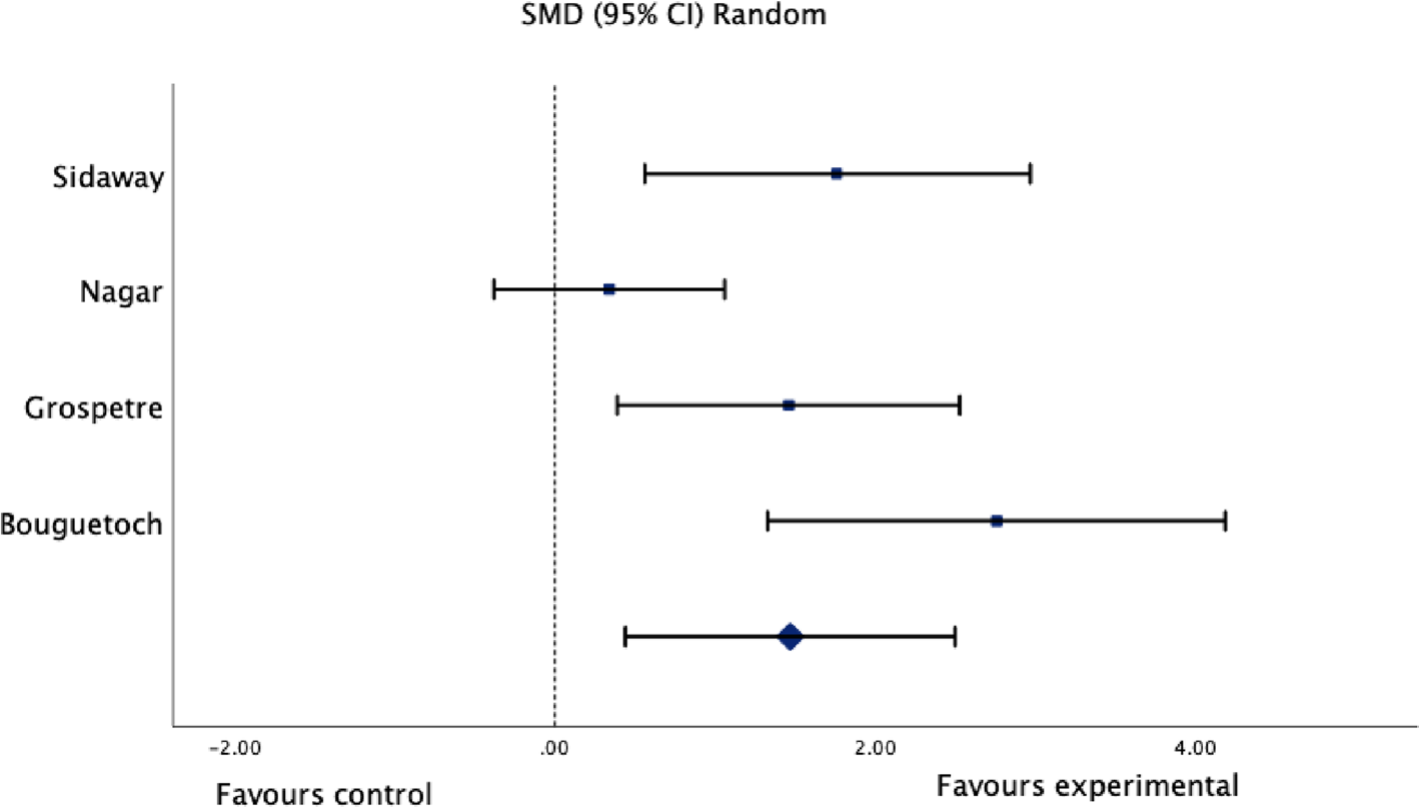
Standardized mean difference (95% CI) in the effect of motor imagery versus control groups on lower leg strength

**Fig. 3:**
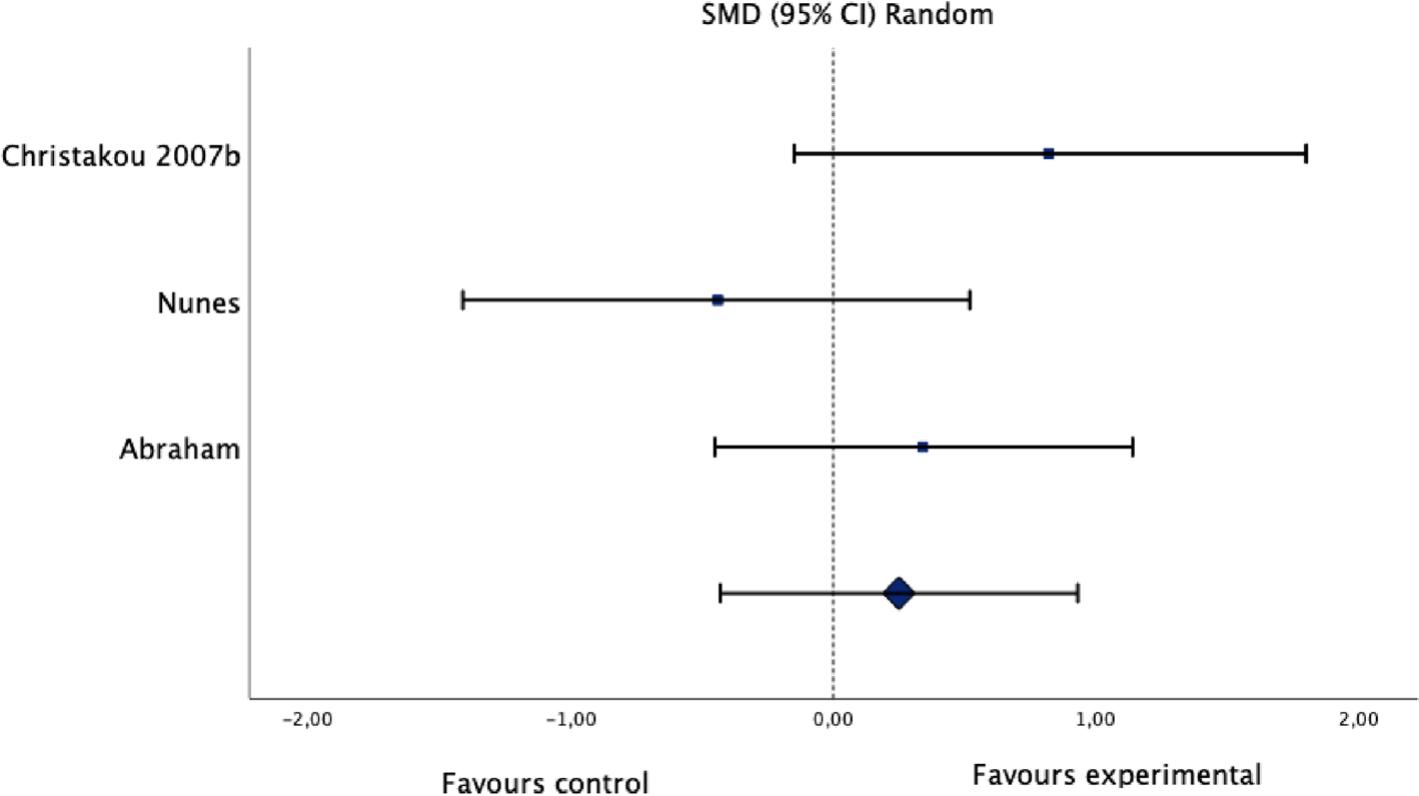
Standardized mean difference (95% CI) in the effect of motor imagery versus control groups on ankle range of motion

**Fig. 4:**
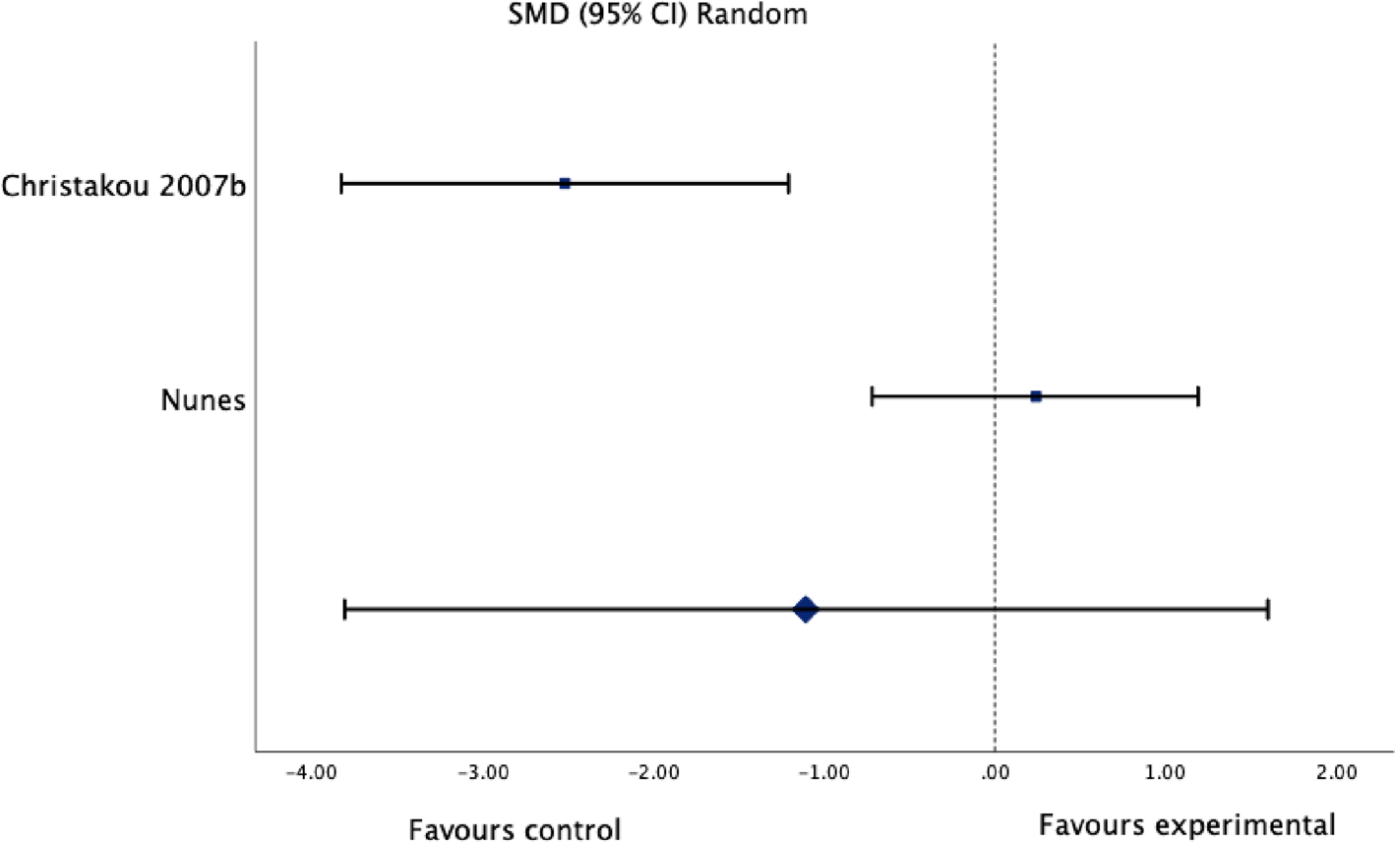
Standardized mean difference (95% CI) in the effect of motor imagery versus control groups on ankle joint edema

**Fig. 5:**
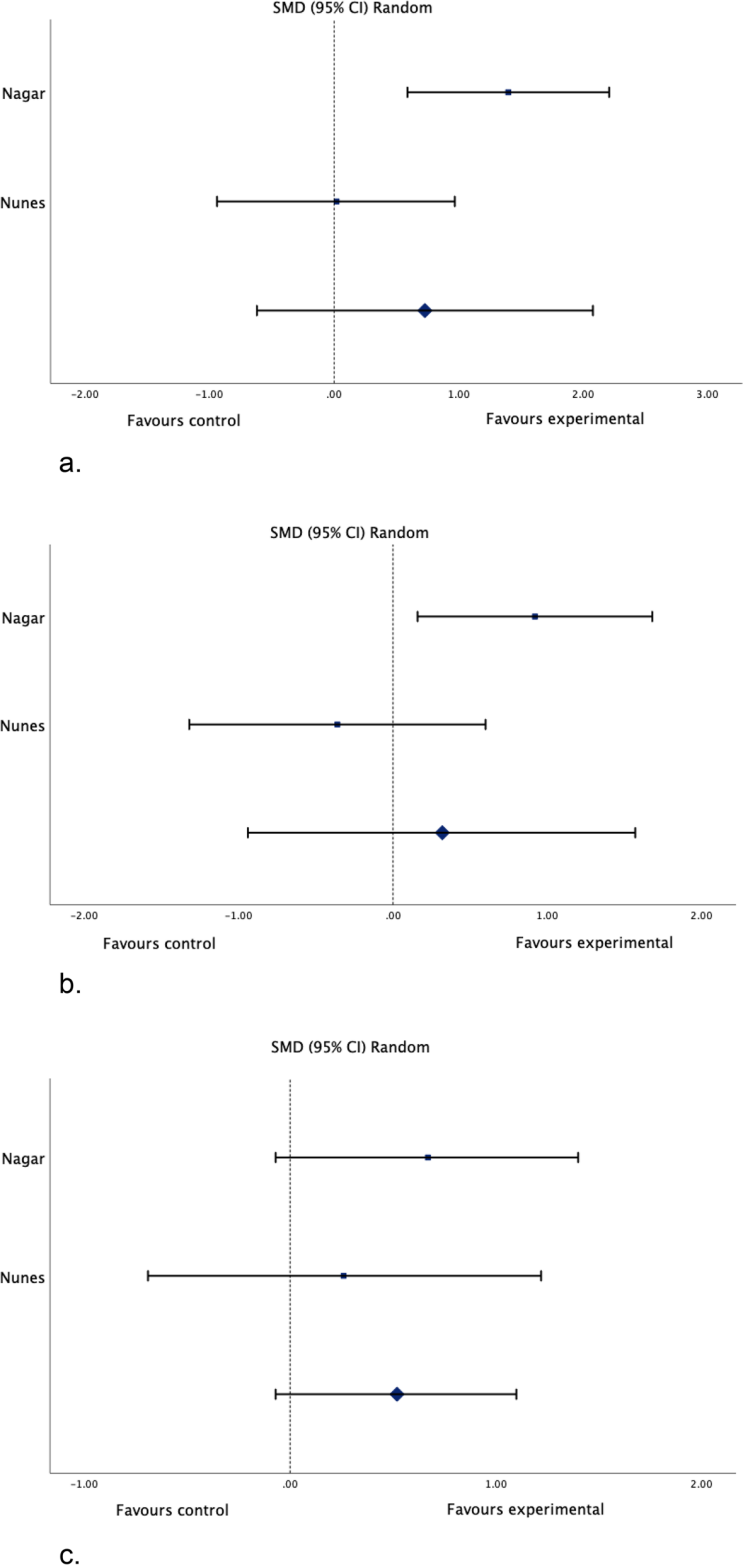
**a** Standardized mean difference (95% CI) in the effect of motor imagery versus control groups on balance (m-SEBT, anterior direction). **b** Standardized mean difference (95% CI) in the effect of motor imagery versus control groups on balance (m-SEBT, posterolateral direction). **c** Standardized mean difference (95% CI) in the effect of motor imagery versus control groups on balance (m-SEBT, posteromedial direction)

Meta-analysis was performed for studies that compared motor imagery to control groups for lower leg strength, ankle range of motion, edema, and balance. One author [64] was contacted, and further information on the change from baseline scores was provided. Results of the meta-analysis can be found in Figs. 2, 3, 4 and 5. Results for the assessment of the certainty of the body of evidence are presented in Table 4.

**Table 4:** Grades of Recommendation, Assessment, Development and Evaluation (GRADE) of the certainty of the body of evidence

Outcome	Trials (n)	Participants	SMD (95% CI), I^2^	Certainty of evidence (GRADE)
Lower leg strength	4	81	1.47 (0.44, 2.50), 73%	Very low certainty^a^
Ankle range of motion	3	60	0.25 (-0.43, 0.93), 41%	Very low certainty^b^
Edema	2	35	1.11 (-1.60, 3.81), 91%	Very low certainty^a^
Balance (SEBT-ant)	2	47	0.73 (-0.62, 2.08), 79%	Very low certainty^a^
Balance (SEBT-PL)	2	47	0.32 (-0.94, 1.57), 76%	Very low certainty^a^
Balance (SEBT-PM)	2	47	0.52 (-0.07, 1.10), 0%	Low certainty^c^

*SMD* standardized mean difference, a: downgraded due to study limitations, imprecision, and substantial heterogeneity, b: downgraded due to study limitations, imprecision, and moderate heterogeneity, c: downgraded due to study limitations, and imprecision

#### Effect of motor imagery on strength

Meta-analysis of four studies (Fig. 2) with a total of 64 healthy participants showed very low certainty evidence of a large, significant effect of motor imagery on lower leg strength when compared to controls (SMD 1.47, 95% CI: 0.44 to 2.50; I^2^: 73%; *p* = 0.005). The evidence was downgraded to very low certainty due to substantial heterogeneity (I^2^ = 73%), limitations in the studies (some concerns in risk of bias in all studies), and imprecision (*n* =  < 300) (Table 4).

#### Effect of motor imagery on range of motion

Meta-analysis of three studies (Fig. 3) with a total of 60 participants (35 subjects with a lateral ankle sprain, and 25 healthy subjects) demonstrated very low certainty evidence that motor imagery, when compared to control groups, had a small non-significant effect on ankle range of motion (SMD 0.25, 95% CI: -0.43 to 0.93; I^2^: 41%; *p* = 0.47). The evidence was downgraded to very low certainty due to moderate heterogeneity (I^2^ = 42%), limitations in the studies (high risk of bias in one study and some concerns for risk of bias in two studies), indirectness (due to differences in study populations and interventions), and imprecision (*n* =  < 300).

#### Effect of motor imagery on edema

Meta-analysis of two studies (Fig. 4) with a total of 35 participants with a lateral ankle sprain provided very low certainty evidence of a non-significant effect on edema (SMD -1.11, 95% CI: -3.82 to 1.60; I^2^: 91%; *p* = 0.42). The evidence was downgraded to very low certainty due to considerable heterogeneity (I^2^ = 91%), limitations in the two studies (high risk of bias in one study and some concerns for risk of bias in the other study), indirectness (due to differences in the intervention), and imprecision (*n* =  < 300).

#### Effect of motor imagery on balance

Meta-analysis of two studies (Fig. 5a) with a total of 47 participants (17 subjects with a lateral ankle sprain, and 30 healthy subjects) demonstrated very low certainty evidence of a moderate, non-significant effect of motor imagery when compared to controls on the anterior direction of the modified star balance excursion test (SMD 0.73, 95% CI: -0.62 to 2.08; I^2^: 79%; *p* = 0.29), very low certainty evidence of a small, non-significant effect of motor imagery on the posterolateral direction (SMD 0.32, 95% CI: -0.94 to 1.57; I^2^: 76%; *p* = 0.62) (Fig. 5b), and low certainty evidence of a moderate, non-significant effect of motor imagery on the posteromedial direction (SMD 0.52, 95% CI: -0.07 to 1.10; I^2^: 0%; *p* = 0.08) (Fig. 5c) of the modified Star Balance Excursion Test. The evidence was downgraded to very low certainty due to limitations in the studies (some concerns for risk of bias in both studies), imprecision (*n* =  < 300), indirectness (due to differences in the study population and intervention), and considerable heterogeneity for the anterior and posterolateral direction (I^2^ = 79% and 76%).

## Discussion

This systematic review with meta-analysis provides an overview of the evidence on the effect of motor imagery on ankle strength, range of motion, balance, and edema in persons with, or without, a lateral ankle sprain. Very low-certainty evidence for a significant, positive effect for motor imagery being able to improve lower leg muscle strength in healthy participants was found. The evidence for balance, ankle range of motion and edema in healthy and injured participants was uncertain, and of very low certainty.

Several methodological strengths for this systematic review can be defined: the protocol was designed using the Cochrane Handbook for Systematic Reviews [65] and Preferred Reporting Items for Systematic reviews and Meta-Analysis (The PRISMA statement) [33]; a focused review question was formulated; a thorough systematic literature search of multiple databases was conducted; only randomized controlled trials were included; the body of evidence was rated using the GRADE criteria; and the motor imagery interventions were rated using the criteria of a successful motor imagery intervention outcome [46]. The assessment of the quality of the motor imagery intervention provides a clear overview of the content and background of the motor imagery intervention (see Table 3). Furthermore, it might give an indication about the chance of finding a positive result.

This study has several limitations. A weakness of the current systematic review and meta-analysis is the small number of studies (*n* = 9) with heterogeneous backgrounds resulting in a limited number of studies and participants per outcome measure. The small number of studies could have underpowered the results. Moreover, certainty of evidence was low to very low because of moderate to high risk-of-bias in the included studies. The risk-of bias assessment showed problems in the blinding of the assessors performing the pre- and post-measurements which could have led to performance bias. Further, no placebo interventions were given to the control groups. A well-developed placebo is hard to construct in motor imagery study designs, however, several published randomized controlled trials in other areas than the ankle joint have used a placebo intervention [28, 67–69]. For instance, a study in persons with an anterior cruciate ligament reconstruction used a neutral task, e.g., mental calculation, or crosswords and showed increased muscle activation of the vastus medialis after five weeks of motor imagery when compared to the mental calculation task in the control group [28]. In a study in persons with non-specific chronic neck pain, mixed results for motor imagery on mobility tasks were found between a motor imagery group, an action observation group, and a placebo action observation group. The intervention groups imagined movement (motor imagery group) and watched a video of the same motor task (action observation group), and the placebo control group watched a video that showed nature landscapes, without any human motor actions [67].

Although caution must be exercised in interpreting the results of the current meta-analysis, the pooled results (*n* = 81, 4 studies) revealed a large, significant effect for motor imagery being able to improve lower leg strength in healthy participants (SMD 1.47; 95% CI: 0.44–2.50), i.e., the plantar and dorsal flexor muscles. The quality of the motor imagery intervention according to criteria of Schuster et al. [46] was rated 10 out of 15 points in two studies [61, 64], and 12 out of 15 points in the other two studies [29, 60]. To obtain a higher quality rating the studies of Grosprêtre et al., Bouguetoch et al., and Sidaway & Trzaska could have added physical practice sessions to their motor imagery intervention and lengthened the duration of the intervention [29, 61, 64]. However, it is questionable whether these modifications would have led to different results as it was previously noted that motor imagery might enhance strength after only a few sessions [25]. Only a small additional increase in strength is expected from a longer training period. In a 4-week training study in upper extremity strength, the major improvement in strength was observed in the first week after four motor imagery and strength training sessions, yet the increase in strength continued over the 4-week period [70].

Further, some differences in comparisons across the included studies were noted: one study added motor imagery to a strength training intervention [60], while the other three studies used a control group who did not participate in any physical or mental activity [29, 61, 64]. In addition, the sample of participants (n = 81) included in the meta-analysis for lower leg strength consisted entirely of young (18 to 26 years), healthy participants, and generalization towards conditions after an acute injury is not possible. Subgroup analysis to explore differences between the effect of motor imagery in healthy and injured participants would have been interesting as it was previously noted that injured persons sometimes suffer from an impaired possibility to generate motor images [16]. In addition, next to a greater difficulty in generating a visual and kinaesthetic motor image, participants with chronic low back pain were found to need more time to complete visualizing a movement [71]. It is therefore important to explore differences between healthy and injured participants. Unfortunately, due to the small number of studies in the meta-analysis, further exploration of subgroups with statistical tests was not possible.

It is important to check for the ability of participants to imagine movements, as the effectiveness of imagery is dependent on the individual capability to generate and control vivid images [72]. Over the last century, various assessment instruments have been developed to assess the imagery ability of an individual. According to a recent published systematic review, the MIQ-R offers sufficient psychometric properties to assess motor imagery ability [73]. In this study the MIQ-R has been used in half of the included articles, and the VMIQ in two others. The VMIQ-2 has the same level of psychometric properties as the MIQ-R, yet the first version of the instrument was used in the studies. The results indicate that for most studies, imagery ability was good. However, in three studies the imagery ability was not assessed, and the topic was not discussed.

The positive effect of motor imagery on lower leg strength found in the current systematic review is in line with results from several recent systematic reviews which studied the effect of motor imagery on strength, but in other body regions than the ankle [20, 74, 75]. In one of these systematic reviews, a large, positive effect with moderate certainty evidence of motor imagery increasing knee extensor strength was demonstrated in participants following a total knee arthroplasty [74].

The pooled results (*n* = 60, 3 studies) of the meta-analysis for ankle range of motion revealed no association with (SMD 0.25; 95% CI: -0.43–0.93) motor imagery improving range of motion of the ankle compared to a control group. An important difference between the three studies can be found in the content of the motor imagery interventions. Especially, the study of Nunes et al. [30] differed greatly from the other studies: a computer showed 40 left–right images (lateralization) of the ankle, the total duration was approximately 2:40 min, the motor imagery was not motor focused, and no detailed and acoustic instruction was given. Therefore only 3 out of 15 criteria for a successful motor imagery intervention were met (see Table 3). The study of Abraham et al. [63] (10/15 criteria) provided a group intervention, did not combine it with physical practice, participants were free to have their eyes open and was applied twice a week [63], despite evidence suggesting better effects when the exercise is done individually, combined with physical practice, with participants having their eyes closed in a quiet place and provided three times per week [46].

Other systematic reviews studying different populations in other regions of the body have found contradictory evidence for motor imagery [20, 74–76]. For instance, a meta-analysis in persons with various musculoskeletal conditions, such as shoulder, knee, and ankle disorders, found no significant effect in acute musculoskeletal conditions on range of motion [76]. Another review stated that the effect of adding motor imagery to standard therapy on active range of motion in patients with a total knee arthroplasty was unclear [75]. Both studies are in line with the results of the current systematic review and do not show a clear effect in acute injuries [75, 76].

With respect to balance, the body of evidence (*n* = 47, 2 studies) showed no association, with only a trend for the posteromedial direction on the Star Balance Excursion Test. When comparing the motor imagery interventions between the two included studies in the meta-analysis for balance, heterogeneity is observed. Nagar & Noohu [60] scored positive on 12 out of 15 criteria for the quality of the intervention, while Nunes et al. [30] scored positive on only 3/15 criteria (see Table 3). This large difference might explain some of the variation between the results of both studies. Other recent systematic reviews, yet directed at different populations, found low certainty, small to moderate effects of action observation and motor imagery on balance [20, 77]. However, those reviews did not use the Star Balance Excursion test as a measurement instrument for balance, but used the Tinetti test [20], Berg Balance Scale, Functional Reach Test, body sway, or rated balance functionally during an obstacle course [77]. Direct comparison of the results of the current meta-analysis with those reviews is therefore limited.

Regarding edema (*n* = 35, 2 studies), no association with motor imagery was found (SMD 1.11; 95% CI: -1.60 to 3.82). The results were classified as indicating very low- certainty evidence. Only two studies could be included in the meta-analysis, again the large effect in favour of the control group in the study of Nunes et al. [30] had a strong influence on the overall effect. A search for other studies evaluating the effect of motor imagery on edema in the field of musculoskeletal disorders resulted in no hits, therefore, a further exploration of this result was not possible.

Due to the very low certainty of the evidence, the effect of motor imagery on muscle strength, ankle range of motion, balance, and edema, in persons with and without a lateral ankle sprain is still uncertain. It is recommended that researchers undertake more high-quality studies with larger sample sizes. Studies should use a randomized controlled trial design with blinded assessors during pre- and post-intervention measurements to decrease the changes of performance bias. Placebo motor imagery intervention should be developed to at least blind the participants, and the motor imagery intervention should score positive on as many of the 15 criteria of Schuster et al. as possible [46]. The criteria from Table 3 provide a framework for the design of a successful motor imagery intervention, and it is likely that a high number of fulfilled criteria results in a higher-quality motor imagery intervention. Further, pre-specified analysis plans should be published to promote unbiased assessment of the data and studies should aim at recruiting athletes with lateral ankle sprains. Researchers, as well as practitioners, are encouraged to use the criteria in developing future motor imagery interventions. Clinicians could use the framework that is discussed in this study as a guideline.

### Supplementary Information

**Additional file 1.** Supplementary material.

## Data Availability

All data generated or analysed during this study are included in this published article and its supplementary information files.
