# Supplementary material for: The effect of movement representation techniques on ankle function and performance in persons with or without a lateral ankle sprain: a systematic review and meta-analysis
Source: BMC Musculoskelet Disord. 2023 Oct 4;24:786. doi: 10.1186/s12891-023-06906-9 (PMC10548724; doi:10.1186/s12891-023-06906-9)
Supplement: Supplementary file 1 — Additional file 1. Supplementary material. [file 12891_2023_6906_MOESM1_ESM.docx]

**Supplementary material**

**Appendix 1. Detailed search strategy**

**MEDLINE**

#1 MH "Ankle" OR MH "Ankle Fractures" OR MH "Ankle Joint" OR MH "Ankle Injuries" OR MH "Arthroplasty, Replacement, Ankle" OR MH "Lateral Ligament, Ankle" OR TI (ankle* OR (talocrural N1 joint*) OR (subtalar N1 joint*) OR (inferior N1 tibiofibular N1 joint*) OR (lateral N1 ligament*) OR (anterior talofibular ligament*) OR (posterior talofibular ligament*) OR (calcaneofibular ligament*)) OR AB (ankle* OR (talocrural N1 joint*) OR (subtalar N1 joint*) OR (inferior N1 tibiofibular N1 joint*) OR (lateral N1 ligament*) OR (anterior talofibular ligament*) OR (posterior talofibular ligament*) OR (calcaneofibular ligament*)) OR SU (ankle* OR (talocrural N1 joint*) OR (subtalar N1 joint*) OR (inferior N1 tibiofibular N1 joint*) OR (lateral N1 ligament*) OR (anterior talofibular ligament*) OR (posterior talofibular ligament*) OR (calcaneofibular ligament*))

#2 TI ((motor N1 imager*) OR (mental N1 imager*) OR (mental N1 practice*) OR (locomotor N1 imager*) OR (visual N1 imager*) OR (guided N1 imager*) OR (kinesthetic N1 imager*) OR (mental N1 training) OR (movement N1 representation N1 technique*) OR imaginat* OR (mental N1 representation*) OR (action N1 (observation* OR training)) OR (mirror N1 therap*)) OR AB ((motor N1 imager*) OR (mental N1 imager*) OR (mental N1 practice*) OR (locomotor N1 imager*) OR (visual N1 imager*) OR (guided N1 imager*) OR (kinesthetic N1 imager*) OR (mental N1 training) OR (movement N1 representation N1 technique*) OR imaginat* OR (mental N1 representation*) OR (action N1 (observation* OR training)) OR (mirror N1 therap*)) OR SU ((motor N1 imager*) OR (mental N1 imager*) OR (mental N1 practice*) OR (locomotor N1 imager*) OR (visual N1 imager*) OR (guided N1 imager*) OR (kinesthetic N1 imager*) OR (mental N1 training) OR (movement N1 representation N1 technique*) OR imaginat* OR (mental N1 representation*) OR (action N1 (observation* OR training)) OR (mirror N1 therap*))

S3 S1 AND S2

**COCHRANE**

#1 (ankle* OR ((talocrural OR astragalocrural) NEAR/1 joint*) OR (subtalar NEAR/1 joint*) OR (inferior NEAR/1 tibiofibular NEAR/1 joint*) OR (lateral NEAR/1 ligament*) OR (talofibular NEAR/1 ligament*) OR (calcaneofibular NEAR/1 ligament*)):ti,ab,kw

#2 ((motor NEAR/1 imager*) OR (mental NEAR/1 imager*) OR (mental NEAR/1 practice*) OR (locomotor NEAR/1 imager*) OR (visual NEAR/1 imager*) OR (guided NEAR/1 imager*) OR (kinesthetic NEAR/1 imager*) OR (mental NEAR/1 training) OR (movement NEAR/1 representation NEAR/1 technique*) OR imaginat* OR (mental NEAR/1 representation*) OR (action NEAR/1 (observation* OR training)) OR (mirror NEAR/1 therap*)):ti,ab,kw

#3 #1 AND #2

**EMBASE**

1 'ankle'/exp OR 'ankle prosthesis'/exp OR 'ankle injury'/exp OR (ankle* OR ((talocrural OR astragalocrural) NEAR/1 joint*) OR (subtalar NEAR/1 joint*) OR (inferior NEAR/1 tibiofibular NEAR/1 joint*) OR (lateral NEAR/1 ligament*) OR (talofibular NEAR/1 ligament*) OR (calcaneofibular NEAR/1 ligament*)):ti,ab,kw

2 'guided imagery'/exp OR ((motor NEAR/1 imager*) OR (mental NEAR/1 imager*) OR (mental NEAR/1 practice*) OR (locomotor NEAR/1 imager*) OR (visual NEAR/1 imager*) OR (guided NEAR/1 imager*) OR (kinesthetic NEAR/1 imager*) OR (mental NEAR/1 training) OR (movement NEAR/1 representation NEAR/1 technique*) OR imaginat* OR (mental NEAR/1 representation*) OR (action NEAR/1 (observation* OR training)) OR (mirror NEAR/1 therap*)):ti,ab,kw

3 #1 AND #2

**CINAHL**

#1 MH "Ankle" OR MH "Lateral Ligament, Ankle" OR MH "Ankle Injuries+" OR (MH "Ankle Surgery+" OR MH "Ankle Joint" OR MH "Talus" OR TI (ankle* OR (talocrural N1 joint*) OR (subtalar N1 joint*) OR (inferior N1 tibiofibular N1 joint*) OR (lateral N1 ligament*) OR (anterior talofibular ligament*) OR (posterior talofibular ligament*) OR (calcaneofibular ligament*)) OR AB (ankle* OR (talocrural N1 joint*) OR (subtalar N1 joint*) OR (inferior N1 tibiofibular N1 joint*) OR (lateral N1 ligament*) OR (anterior talofibular ligament*) OR (posterior talofibular ligament*) OR (calcaneofibular ligament*)) OR SU (ankle* OR (talocrural N1 joint*) OR (subtalar N1 joint*) OR (inferior N1 tibiofibular N1 joint*) OR (lateral N1 ligament*) OR (anterior talofibular ligament*) OR (posterior talofibular ligament*) OR (calcaneofibular ligament*))

#2 TI ((motor N1 imager*) OR (mental N1 imager*) OR (mental N1 practice*) OR (locomotor N1 imager*) OR (visual N1 imager*) OR (guided N1 imager*) OR (kinesthetic N1 imager*) OR (mental N1 training) OR (movement N1 representation N1 technique*) OR imaginat* OR (mental N1 representation*) OR (action N1 (observation* OR training)) OR (mirror N1 therap*)) OR AB ((motor N1 imager*) OR (mental N1 imager*) OR (mental N1 practice*) OR (locomotor N1 imager*) OR (visual N1 imager*) OR (guided N1 imager*) OR (kinesthetic N1 imager*) OR (mental N1 training) OR (movement N1 representation N1 technique*) OR imaginat* OR (mental N1 representation*) OR (action N1 (observation* OR training)) OR (mirror N1 therap*)) OR SU ((motor N1 imager*) OR (mental N1 imager*) OR (mental N1 practice*) OR (locomotor N1 imager*) OR (visual N1 imager*) OR (guided N1 imager*) OR (kinesthetic N1 imager*) OR (mental N1 training) OR (movement N1 representation N1 technique*) OR imaginat* OR (mental N1 representation*) OR (action N1 (observation* OR training)) OR (mirror N1 therap*))

S3 S1 AND S2

**SPORTDISCUS**

S1 DE "ANKLE" OR DE "ANKLEBONE" OR DE "ANKLE injuries" OR DE "ANKLE dislocation" OR DE "ANKLE injury treatment" OR DE "ANKLE fractures" OR DE "ANKLE lateral ligament" OR DE "ANKLE physiology" OR DE "PLANTARFLEXION" OR DE "PERONEAL tendons" OR DE "SUPINATION" OR DE "ANKLE injuries" OR DE "FOOT injuries" OR DE "PRONATION" OR TI (ankle* OR (talocrural N1 joint*) OR (subtalar N1 joint*) OR (inferior N1 tibiofibular N1 joint*) OR (lateral N1 ligament*) OR (anterior talofibular ligament*) OR (posterior talofibular ligament*) OR (calcaneofibular ligament*)) OR AB (ankle* OR (talocrural N1 joint*) OR (subtalar N1 joint*) OR (inferior N1 tibiofibular N1 joint*) OR (lateral N1 ligament*) OR (anterior talofibular ligament*) OR (posterior talofibular ligament*) OR (calcaneofibular ligament*)) OR SU (ankle* OR (talocrural N1 joint*) OR (subtalar N1 joint*) OR (inferior N1 tibiofibular N1 joint*) OR (lateral N1 ligament*) OR (anterior talofibular ligament*) OR (posterior talofibular ligament*) OR (calcaneofibular ligament*))

S2 DE "MOTOR imagery (Cognition)" OR TI ((motor N1 imager*) OR (mental N1 imager*) OR (mental N1 practice*) OR (locomotor N1 imager*) OR (visual N1 imager*) OR (guided N1 imager*) OR (kinesthetic N1 imager*) OR (mental N1 training) OR (movement N1 representation N1 technique*) OR imaginat* OR (mental N1 representation*) OR (action N1 (observation* OR training)) OR (mirror N1 therap*)) OR AB ((motor N1 imager*) OR (mental N1 imager*) OR (mental N1 practice*) OR (locomotor N1 imager*) OR (visual N1 imager*) OR (guided N1 imager*) OR (kinesthetic N1 imager*) OR (mental N1 training) OR (movement N1 representation N1 technique*) OR imaginat* OR (mental N1 representation*) OR (action N1 (observation* OR training)) OR (mirror N1 therap*)) OR SU ((motor N1 imager*) OR (mental N1 imager*) OR (mental N1 practice*) OR (locomotor N1 imager*) OR (visual N1 imager*) OR (guided N1 imager*) OR (kinesthetic N1 imager*) OR (mental N1 training) OR (movement N1 representation N1 technique*) OR imaginat* OR (mental N1 representation*) OR (action N1 (observation* OR training)) OR (mirror N1 therap*))

S3 S1 AND S2

**WEB OF SCIENCE**

#1 TS=(ankle* OR ((talocrural OR astragalocrural) NEAR/1 joint*) OR (subtalar NEAR/1 joint*) OR (inferior NEAR/1 tibiofibular NEAR/1 joint*) OR (lateral NEAR/1 ligament*) OR (talofibular NEAR/1 ligament*) OR (calcaneofibular NEAR/1 ligament*))

#2 TS=((motor NEAR/1 imager*) OR (mental NEAR/1 imager*) OR (mental NEAR/1 practice*) OR (locomotor NEAR/1 imager*) OR (visual NEAR/1 imager*) OR (guided NEAR/1 imager*) OR (kinesthetic NEAR/1 imager*) OR (mental NEAR/1 training) OR (movement NEAR/1 representation NEAR/1 technique*) OR imaginat* OR (mental NEAR/1 representation*) OR (action NEAR/1 (observation* OR training)) OR (mirror NEAR/1 therap*))

#3 #1 AND #2

**PSYCINFO**

S1 DE "Ankle" OR TI (ankle* OR (talocrural N1 joint*) OR (subtalar N1 joint*) OR (inferior N1 tibiofibular N1 joint*) OR (lateral N1 ligament*) OR (anterior talofibular ligament*) OR (posterior talofibular ligament*) OR (calcaneofibular ligament*)) OR AB (ankle* OR (talocrural N1 joint*) OR (subtalar N1 joint*) OR (inferior N1 tibiofibular N1 joint*) OR (lateral N1 ligament*) OR (anterior talofibular ligament*) OR (posterior talofibular ligament*) OR (calcaneofibular ligament*)) OR SU (ankle* OR (talocrural N1 joint*) OR (subtalar N1 joint*) OR (inferior N1 tibiofibular N1 joint*) OR (lateral N1 ligament*) OR (anterior talofibular ligament*) OR (posterior talofibular ligament*) OR (calcaneofibular ligament*))

S2 DE "Guided Imagery" OR TI ((motor N1 imager*) OR (mental N1 imager*) OR (mental N1 practice*) OR (locomotor N1 imager*) OR (visual N1 imager*) OR (guided N1 imager*) OR (kinesthetic N1 imager*) OR (mental N1 training) OR (movement N1 representation N1 technique*) OR imaginat* OR (mental N1 representation*) OR (action N1 (observation* OR training)) OR (mirror N1 therap*)) OR AB ((motor N1 imager*) OR (mental N1 imager*) OR (mental N1 practice*) OR (locomotor N1 imager*) OR (visual N1 imager*) OR (guided N1 imager*) OR (kinesthetic N1 imager*) OR (mental N1 training) OR (movement N1 representation N1 technique*) OR imaginat* OR (mental N1 representation*) OR (action N1 (observation* OR training)) OR (mirror N1 therap*)) OR SU ((motor N1 imager*) OR (mental N1 imager*) OR (mental N1 practice*) OR (locomotor N1 imager*) OR (visual N1 imager*) OR (guided N1 imager*) OR (kinesthetic N1 imager*) OR (mental N1 training) OR (movement N1 representation N1 technique*) OR imaginat* OR (mental N1 representation*) OR (action N1 (observation* OR training)) OR (mirror N1 therap*))

S3 S1 AND S2

**GOOGLE SCHOLAR**

Ankle AND (“motor imagery” OR “mental imagery” )

OR “mental practice” OR “locomotor imagery” OR “visual imagery” OR “kinesthetic imagery” OR “mental training” OR “movement representation technique” OR imagination OR “mental representation” OR “action observation training” OR “mirror therapy”)

**Appendix 2. Inclusion criteria based on PICOS**

Study design

- Randomized Controlled Trials

Population

- Healthy participants or participants with a lateral ankle sprain

Intervention

- Movement representation techniques (motor imagery or action observation) in isolation, or in combination with usual care.

Control

- Usual care, or an intervention which differed from the movement representation techniques or a placebo intervention

Outcomes

- Muscle strength, muscle endurance, range of motion, balance, return to sports tests, or questionnaires on self-reported function or activities

**Appendix 3. Data extraction form**

| **Data extraction form** | **1. Data extracted on:** |
| --- | --- |
| Author(s) |  |
| Year of publication |  |
| Origin/ country of origin |  |
| Aim of the study |  |
| Inclusion criteria |  |
| Exclusion criteria |  |
| **Demographic description of the population:** |  |
| Gender |  |
| Age |  |
| Type of injury |  |
| Duration of injury |  |
| Sample size |  |
| Methodology |  |
| **Intervention:** |  |
| Design |  |
| Application and theory |  |
| Duration of the intervention |  |
| Comparison group |  |
| Outcome measures |  |
| Measurement instruments |  |
| Validity of measurement instruments |  |
| Reliability of measurement instruments |  |
| Follow-up (and duration) |  |
| Key findings |  |
| Reference to other relevant studies |  |
| Correspondence required |  |

**Appendix 4. Number of identified records per database**

29-03-2021 24-02-2022 07-06-2023

Cinahl 29 31 32

Cochrane 41 53 65

Embase 105 118 119

Medline 69 74 83

PsycInfo 29 29 30

Scholar 210* 200* 200*

SportDiscus 28 29 30

WOS 74 77 80

*the difference in results is due to the algorithm of Google.

**Appendix 5. Number of potential identified trials**

Trial register No. No. relevant

ISRCTN: 12 0

WHO ICTRP: 3 2

Clinical Trials Register EU 2 0

Clinical Trials GOV 112 0

Trial Register Netherlands 205 0

**Appendix 6. Detailed forest plots**


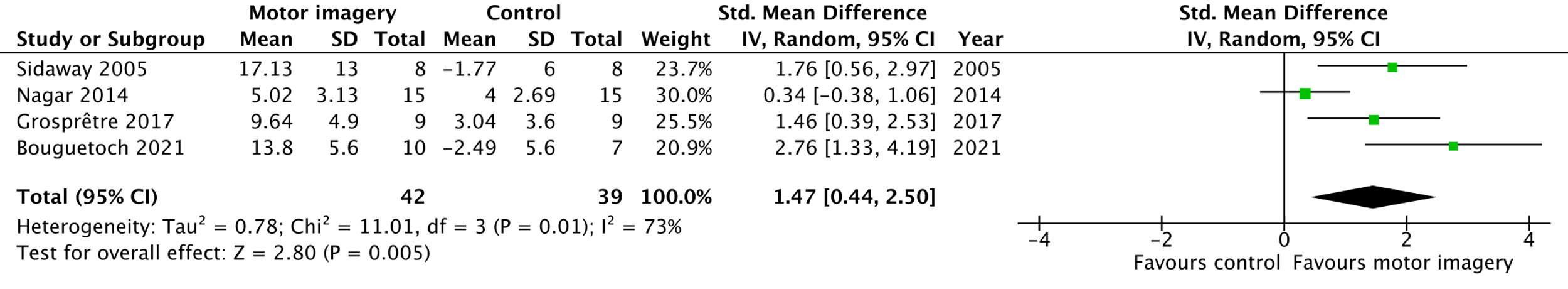


Figure S1. Standardized mean difference (95% CI) in the effect of motor imagery versus control groups on lower leg strength.


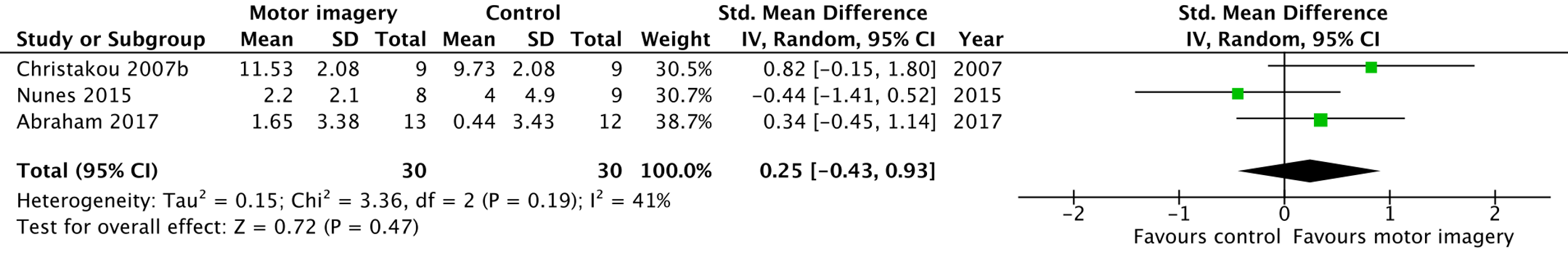


Figure S2. Standardized mean difference (95% CI) in the effect of motor imagery versus control groups on ankle range of motion.


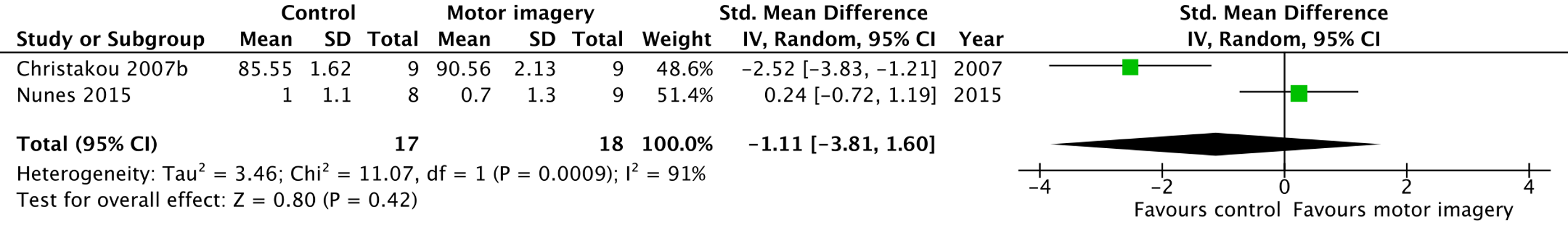


Figure S3. Standardized mean difference (95% CI) in the effect of motor imagery versus control groups on ankle joint edema.


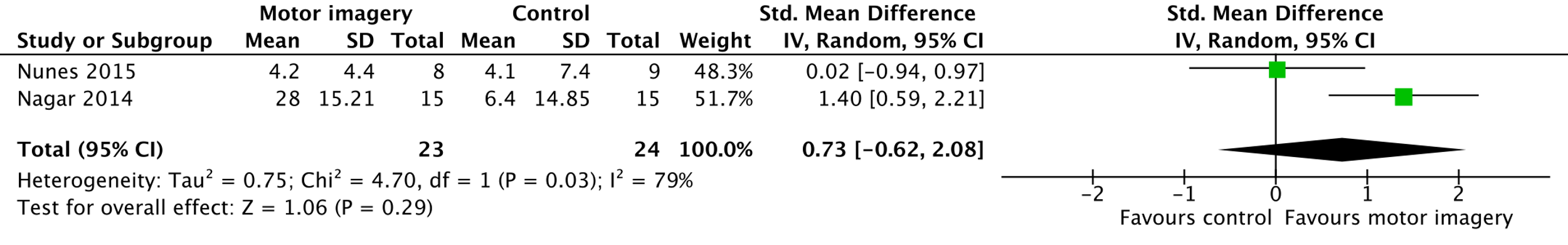


Figure S4. Standardized mean difference (95% CI) in the effect of motor imagery versus control groups on balance (m-SEBT, anterior direction).


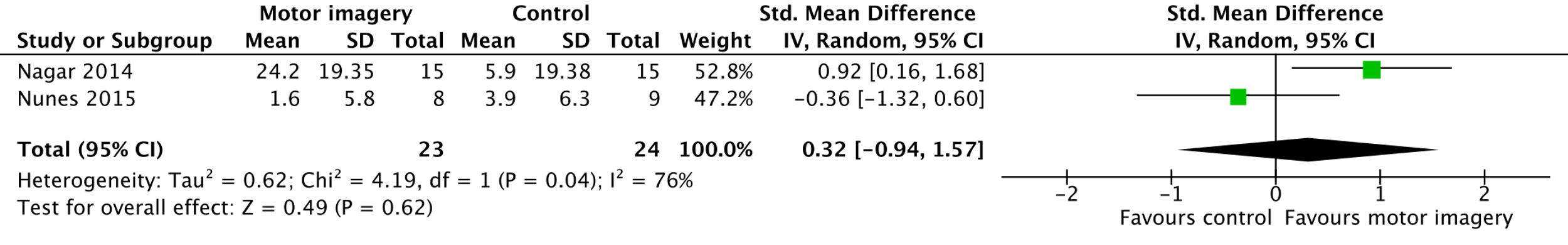


Figure S5. Standardized mean difference (95% CI) in the effect of motor imagery versus control groups on balance (m-SEBT, posterolateral direction).


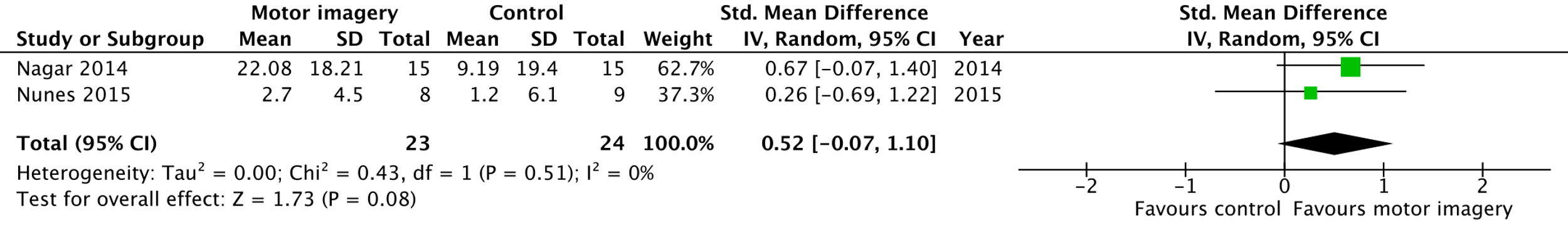


Figure S6. Standardized mean difference (95% CI) in the effect of motor imagery versus control groups on balance (m-SEBT, posteromedial direction).

**Appendix 7. PRISMA checklist**

| **Section and Topic** | **Item #** | **Checklist item** | **Location where item is reported** |
| --- | --- | --- | --- |
| **TITLE** | | |  |
| Title | 1 | Identify the report as a systematic review. | Line 2 |
| **ABSTRACT** | | |  |
| Abstract | 2 | See the PRISMA 2020 for Abstracts checklist. | Lines 33-77 |
| **INTRODUCTION** | | |  |
| Rationale | 3 | Describe the rationale for the review in the context of existing knowledge. | Lines 128-133 |
| Objectives | 4 | Provide an explicit statement of the objective(s) or question(s) the review addresses. | Lines 135-137 |
| **METHODS** | | |  |
| Eligibility criteria | 5 | Specify the inclusion and exclusion criteria for the review and how studies were grouped for the syntheses. | Lines 168-193 |
| Information sources | 6 | Specify all databases, registers, websites, organisations, reference lists and other sources searched or consulted to identify studies. Specify the date when each source was last searched or consulted. | Lines 144-148 and lines 160-161 and lines 229-231 |
| Search strategy | 7 | Present the full search strategies for all databases, registers and websites, including any filters and limits used. | Appendix 2 |
| Selection process | 8 | Specify the methods used to decide whether a study met the inclusion criteria of the review, including how many reviewers screened each record and each report retrieved, whether they worked independently, and if applicable, details of automation tools used in the process. | Lines 144-164 |
| Data collection process | 9 | Specify the methods used to collect data from reports, including how many reviewers collected data from each report, whether they worked independently, any processes for obtaining or confirming data from study investigators, and if applicable, details of automation tools used in the process. | Lines 195-209 |
| Data items | 10a | List and define all outcomes for which data were sought. Specify whether all results that were compatible with each outcome domain in each study were sought (e.g. for all measures, time points, analyses), and if not, the methods used to decide which results to collect. | Lines 191-193 and appendix 3 |
|  | 10b | List and define all other variables for which data were sought (e.g. participant and intervention characteristics, funding sources). Describe any assumptions made about any missing or unclear information. | See appendix 3 |
| Study risk of bias assessment | 11 | Specify the methods used to assess risk of bias in the included studies, including details of the tool(s) used, how many reviewers assessed each study and whether they worked independently, and if applicable, details of automation tools used in the process. | Lines 171-176 |
| Effect measures | 12 | Specify for each outcome the effect measure(s) (e.g. risk ratio, mean difference) used in the synthesis or presentation of results. | Lines 217-222 |
| Synthesis methods | 13a | Describe the processes used to decide which studies were eligible for each synthesis (e.g. tabulating the study intervention characteristics and comparing against the planned groups for each synthesis (item #5)). | Lines 202-209 |
|  | 13b | Describe any methods required to prepare the data for presentation or synthesis, such as handling of missing summary statistics, or data conversions. | Lines 195-222 |
|  | 13c | Describe any methods used to tabulate or visually display results of individual studies and syntheses. | Line 216 |
|  | 13d | Describe any methods used to synthesize results and provide a rationale for the choice(s). If meta-analysis was performed, describe the model(s), method(s) to identify the presence and extent of statistical heterogeneity, and software package(s) used. | Lines 211-222 |
|  | 13e | Describe any methods used to explore possible causes of heterogeneity among study results (e.g. subgroup analysis, meta-regression). | Lines 212-215 and lines 223-227 |
|  | 13f | Describe any sensitivity analyses conducted to assess robustness of the synthesized results. | Lines 222-227 |
| Reporting bias assessment | 14 | Describe any methods used to assess risk of bias due to missing results in a synthesis (arising from reporting biases). | Lines 229-231 |
| Certainty assessment | 15 | Describe any methods used to assess certainty (or confidence) in the body of evidence for an outcome. | Lines 232-237 |
| **RESULTS** | | |  |
| Study selection | 16a | Describe the results of the search and selection process, from the number of records identified in the search to the number of studies included in the review, ideally using a flow diagram. | Lines 254-264 + figure 1 |
|  | 16b | Cite studies that might appear to meet the inclusion criteria, but which were excluded, and explain why they were excluded. | Lines 260-264 |
| Study characteristics | 17 | Cite each included study and present its characteristics. | Line 268 and table 2. |
| Risk of bias in studies | 18 | Present assessments of risk of bias for each included study. | Table 1 and lines 273-285 |
| Results of individual studies | 19 | For all outcomes, present, for each study: (a) summary statistics for each group (where appropriate) and (b) an effect estimate and its precision (e.g. confidence/credible interval), ideally using structured tables or plots. | See table 2 |
| Results of syntheses | 20a | For each synthesis, briefly summarise the characteristics and risk of bias among contributing studies. | Lines 364-407 |
|  | 20b | Present results of all statistical syntheses conducted. If meta-analysis was done, present for each the summary estimate and its precision (e.g. confidence/credible interval) and measures of statistical heterogeneity. If comparing groups, describe the direction of the effect. | Lines 364-407 |
|  | 20c | Present results of all investigations of possible causes of heterogeneity among study results. | Lines 364-407 |
|  | 20d | Present results of all sensitivity analyses conducted to assess the robustness of the synthesized results. | N/A – not enough studies in synthesis |
| Reporting biases | 21 | Present assessments of risk of bias due to missing results (arising from reporting biases) for each synthesis assessed. | N/A – not enough studies in synthesis |
| Certainty of evidence | 22 | Present assessments of certainty (or confidence) in the body of evidence for each outcome assessed. | Lines 364-407 |
| **DISCUSSION** | | |  |
| Discussion | 23a | Provide a general interpretation of the results in the context of other evidence. | Lines 409-414 |
|  | 23b | Discuss any limitations of the evidence included in the review. | Lines 446-482 |
|  | 23c | Discuss any limitations of the review processes used. | Lines 427-444 |
|  | 23d | Discuss implications of the results for practice, policy, and future research. | Lines 537-548 |
| **OTHER INFORMATION** | | |  |
| Registration and protocol | 24a | Provide registration information for the review, including register name and registration number, or state that the review was not registered. | Line 76 and line 141 |
|  | 24b | Indicate where the review protocol can be accessed, or state that a protocol was not prepared. | N/A |
|  | 24c | Describe and explain any amendments to information provided at registration or in the protocol. | Title has been updated. Outcome measures have been updated. |
| Support | 25 | Describe sources of financial or non-financial support for the review, and the role of the funders or sponsors in the review. | No funding provided |
| Competing interests | 26 | Declare any competing interests of review authors. | No competing interests |
| Availability of data, code and other materials | 27 | Report which of the following are publicly available and where they can be found: template data collection forms; data extracted from included studies; data used for all analyses; analytic code; any other materials used in the review. | Template data collection form can be found in the supplementary files. So is the search strategy, a detailed overview of the number of identified records per database and detailed forest plots.  Additional information is available on request. |

*From:*  Page MJ, McKenzie JE, Bossuyt PM, Boutron I, Hoffmann TC, Mulrow CD, et al. The PRISMA 2020 statement: an updated guideline for reporting systematic reviews. BMJ 2021;372:n71. doi: 10.1136/bmj.n71

For more information, visit: <http://www.prisma-statement.org/>
